# Specific phosphorylation of the *Pf*Rh2b invasion ligand of *Plasmodium falciparum*

**DOI:** 10.1042/BJ20121694

**Published:** 2013-05-31

**Authors:** Klemens Engelberg, Aditya S. Paul, Boris Prinz, Maya Kono, Wilhelm Ching, Dorothee Heincke, Thomas Dobner, Tobias Spielmann, Manoj T. Duraisingh, Tim-Wolf Gilberger

**Affiliations:** *Bernhard-Nocht-Institute for Tropical Medicine, Department of Molecular Parasitology, Hamburg, Germany; †Harvard School of Public Health, Department of Immunology and Infectious Diseases, Boston, U.S.A.; ‡Heinrich Pette Institute, Leibniz Institute for Experimental Virology, Department of Molecular Virology, Hamburg, Germany; §M.G. DeGroote Institute for Infectious Disease Research and Department of Pathology and Molecular Medicine, McMaster University, Hamilton, Canada

**Keywords:** casein kinase 2 (CK2), malaria, phosphorylation, *Plasmodium falciparum*, red blood cell invasion, reticulocyte homologue protein 2b (Rh2b), AMA1, apical membrane antigen 1, CDPK1, calcium-dependent protein kinase 1, CK2, casein kinase 2, CPD, cytoplasmic domain, DAPI, 4′,6-diamidino-2-phenylindole, DTT, dithiothreitol, EBA, erythrocyte-binding antigen, EBL, erythrocyte binding-like, gDNA, genomic DNA, GFP, green fluorescent protein, GST, glutathione transferase, HA, haemagglutinin, hDHFR, human dihydrofolate reductase, HRP, horseradish peroxidase, IFA, immunofluorescence assay, *Mm*PKAc, *Mus musculus* protein kinase A catalytic subunit, *Pf*CK2, *Plasmodium falciparum* CK2, PKA, protein kinase A, PKC, protein kinase C, PMP, protein metallophosphatase, Rh, reticulocyte homologue protein, TBCA, tetrabromocinnamic acid, *Zm*CK2α, *Zea mays* CK2 α subunit

## Abstract

Red blood cell invasion by the malaria parasite *Plasmodium falciparum* relies on a complex protein network that uses low and high affinity receptor–ligand interactions. Signal transduction through the action of specific kinases is a control mechanism for the orchestration of this process. In the present study we report on the phosphorylation of the CPD (cytoplasmic domain) of *P. falciparum* Rh2b (reticulocyte homologue protein 2b). First, we identified Ser^3233^ as the sole phospho-acceptor site in the CPD for *in vitro* phosphorylation by parasite extract. We provide several lines of evidence that this phosphorylation is mediated by *Pf*CK2 (*P. falciparum* casein kinase 2): phosphorylation is cAMP independent, utilizes ATP as well as GTP as phosphate donors, is inhibited by heparin and tetrabromocinnamic acid, and is mediated by purified *Pf*CK2. We raised a phospho-specific antibody and showed that Ser^3233^ phosphorylation occurs in the parasite prior to host cell egress. We analysed the spatiotemporal aspects of this phosphorylation using immunoprecipitated endogenous Rh2b and minigenes expressing the CPD either at the plasma or rhoptry membrane. Phosphorylation of Rh2b is not spatially restricted to either the plasma or rhoptry membrane and most probably occurs before Rh2b is translocated from the rhoptry neck to the plasma membrane.

## INTRODUCTION

The most serious and potentially fatal type of malaria is caused by *Plasmodium falciparum*. Transmission of this protozoan pathogen occurs during feeding of an Anopheles mosquito. After an initial multiplication step in liver cells, the parasite invades erythrocytes and multiplies by a process known as schizogony within red blood cells. Rupture of a red blood cell marks the end of multiplication followed by re-invasion into new erythrocytes [1]. This intracellular part of the asexual life cycle takes 48 h for *P. falciparum* parasites to complete. In contrast, the invasion of erythrocytes by invasive ‘merozoite’ forms is a rapid [2–4] and complex process that relies on an orchestrated cascade of interactions between the invading parasite and host cells [5].

After the initial attachment of the parasite to the surface of the target cell, the parasite establishes a tight junction between its apical end and the host-cell membrane. This tight junction progressively moves towards the posterior end of the invading parasite as it enters a red blood cell [6]. This process is driven by the intracellular translocation machinery of the parasite involving transmembrane proteins connected to the actinomyosin contractile system [7]. An array of proteins that play an important part in invasion is located in specialized exocytic organelles (micronemes, rhoptries and dense granules) [8].

Upon secretion from these organelles, some of these proteins function as cellular adhesins that mediate host-cell penetration and subsequent establishment within the red blood cell [9].

The most extensively studied proteins belong to the EBL (erythrocyte binding-like) protein family, RBL (reticulocyte binding-like homologues)/Rh (reticulocyte homologue protein), the TRAP (thrombospondin anonymous protein) family AMA1 (apical membrane antigen 1). Whereas most of these parasite adhesins interact with erythrocyte surface receptors [10–16], the extracellular domain of AMA1 forms a complex with proteins inserted by the parasite into the erythrocyte membrane [17]. Although much smaller in size than the ectodomains and less obvious in their molecular functions, the short CPD (cytoplasmic domain) of these adhesins appears to be essential for their function [3,18–20].

For the EBL protein EBA (erythrocyte binding antigen)-175, the CPD is dispensable for proper trafficking, but essential for a functional EBA-175/glycophorin A pathway of invasion [19]. For the Rh protein Rh2b, deletion of the entire cytoplasmic tail or mutation of a conserved acidic motif within the tail is not tolerated by the parasite [21]. Additionally, exchange of the CPD of Rh2a with the Rh2b CPD stimulates the activity of the otherwise inactive parasite ligand [20]. Although this finding demonstrates a specific function of the CPD, the sequence elements within this domain that distinguishes its functional form from the inactive Rh2a analogue are not known.

For AMA1, deletion of its CPD blocks the parasitic invasion of erythrocytes [3]. Subsequent work revealed functional insights into the pivotal role of the CPD. It showed that AMA1 is phosphorylated, where phosphorylation is mediated by the cAMP-dependent protein kinase [PKA (protein kinase A)]. A serine (Ser^610^) was identified as a critical residue and its phosphorylation was established as essential for invasion [22]. This supported earlier work that highlighted the role of kinases during the invasion process. For instance, CDPK1 (calcium-dependent protein kinase 1) phosphorylates proteins of the actin–myosin motor complex, one known lynchpin for active invasion [23]. In *Toxoplasma gondii*, another apicomplexan parasite, host-cell invasion can be blocked by the inhibition of CK2 (casein kinase 2) [24]. This is consistent with a recent publication that has implicated the interaction of the regulatory subunit β1 of CK2 with proteins involved in invasion and the motility of the malaria parasite [25]. Although the role of CPD phosphorylation of AMA1 is well established, nothing is known about the roles of CPD phosphorylation in other adhesins. The present study identifies a specific phosphorylation event in the Rh2b invasion ligand.

## EXPERIMENTAL

### Nucleic acids and constructs

GST (glutathione transferase)–Rh2b fusion was achieved by the amplification of the 3′-end (135 bp excluding the stop codon) of *rh2b* (PlasmoDB accession number PF3D7_1335300) from 3D7 gDNA (genomic DNA) and cloned into BamHI and XhoI restriction sites of the pGEX-4T-1 vector (GE Healthcare). Recombinant GST fusion proteins were purified according to the manufacturer's protocol (GE Healthcare). Mutations of the Rh2b CPD were introduced by using a two-step primer-directed PCR mutagenesis method with proof reading Phusion polymerase (New England Biolabs). All oligonucleotides used in the present study are listed in Supplementary Table S1 (at http://www.biochemj.org/bj/452/bj4520457add.htm). 3D7 parasites expressing Rh2b as a 3×HA (haemagglutinin) fusion protein were generated by 3′ replacement of the endogenous *rh2b* gene. The last 1113 bp of the Rh2b-encoding region (8649–9762 bp, excluding the stop codon) were amplified from 3D7 gDNA and cloned in the NotI and AvrII restriction sites of the transfection vector pARL 1a-3×HA [22]. NotI/AvrII restriction releases the *ama1* promoter in this vector and generates a 3′ replacement constructs that, only after integration, leads to a detectable Rh2b–HA product. Single cross-over integration was confirmed by PCR.

3D7 parasites expressing the α subunit of CK2 as a 3× HA fusion protein were generated by amplifying the coding region of PlasmoDB accession number PF3D7_1108400 from 3D7 gDNA with subsequent cloning in the KpnI and AvrII restriction sites of the transfection vector pARL 1a-3×HA using AvrII and XbaI compatibility. Transcription of CK2–HA is controlled by the *crt* (chloroquine resistance transporter) promoter. Additionally, the endogenous *ck2* α locus was tagged with HA by 3′ replacement. To achieve this, the open reading frame of *ck2*, without the start and stop codons, was amplified from 3D7 gDNA and cloned in the NotI and AvrII restriction sites of the transfection vector pARL 1a-3×HA using XbaI and AvrII compatibility. Single cross-over integration was confirmed by PCR and expression was analysed by Western blotting and microscopy.

### Parasites strains, transfection and single-cross-over integration

*P. falciparum* at asexual stages were cultured in human O^+^ erythrocytes according to standard procedures [26]. 3D7 parasites were transfected as described previously [27] with 100 μg of purified plasmid DNA. Positive selection for transfected parasites was achieved using 2.5–5 nM WR99210 (Jacobus Pharmaceuticals), an antifolate that selects for the presence of the hDHFR (human dihydrofolate reductase) marker. For single-cross-over integration, following transfection parasites were alternately grown with and without WR99210 pressure (~2– 3 weeks for each interval off-drug) to promote integration into the *rh2b and ck2* locus.

### *In vitro* phosphorylation assay

Parasite lysate used in the *in vitro* phosphorylation assays was generated by separating infected [100 ml of synchronized 3D7 schizont culture with >7% parasitaemia (4% haematocrit)] from uninfected erythrocytes by magnetic sorting using VarioMACS (Militenyi Biotech). After saponin lysis, parasites were lysed in 10 volumes of ice-cold buffer B [50 mM Tris/HCl (pH 7.3), 50 mM β-glycerophosphate, 1 mM DTT (diothiothreitol), Complete™ Protease Inhibitor Cocktail (Roche) and Phosphatase Inhibitor Cocktail I (Sigma)] by several passages through a needle and three cycles of freeze–thaw in liquid nitrogen. The membrane fraction was separated from the soluble proteins by centrifugation at 13000 ***g*** for 30 min at 4°C. Control extracts were made from similar amounts of uninfected red blood cells. Radioactive kinase assays were carried out in 35 μl of standard reactions containing kinase buffer [50 mM Tris/HCl (pH 7.5), 10 mM MgCl_2_ and 1 mM DTT], 25 μM ATP (Sigma) and 1–2 μg of recombinant fusion protein bound on glutathione–Sepharose beads (Genscript). Assays were carried out with or without 10 μM cAMP (Sigma). For kinase assays using recombinant *Mm*PKAc (*Mus musculus* PKA catalytic subunit) or *Zm*CK2α (*Zea mays* CK2 α subunit) (Biaffin), 300 units of kinase were mixed with the indicated amount of recombinant protein, kinase buffer and ATP on ice. Reactions were started with addition of 1 μCi [γ-^32^P]ATP (Hartmann Analytics) and incubated at 30°C for 40 min. Beads were washed three times with ice-cold buffer B and the reaction was terminated by the addition of 5× SDS loading dye with subsequent boiling at 95°C for 3 min. Proteins were separated by SDS/PAGE (15% gel) and stained with SimplyBlue™ Coomassie Safestain (Invitrogen). Finally, the stained gels were shrink-wrapped on 3MM Whatmann paper (Schleicher & Schuell) and autoradiograms were generated using RP NEW medical X-ray screens (CEA). Non-radioactive kinase assays were carried out as described above. Proteins were separated by SDS/PAGE (10% gel) and detected by Western blotting.

For treatment with different inhibitors, heparin (Ratiopharm), TBCA (tetrabromocinnamic acid; Merck) and staurosporine (Merck), samples were mixed with the indicated concentrations of inhibitory molecules and the treated and mock-treated samples were incubated for 5 min at 30°C, stored on ice and mixed with ATP. Reactions were started by incubation at 30°C for 40 min. GTP (Sigma) was used in the same concentration as ATP (25 μM) with the addition of 0.5 mM manganese chloride (New England Biolabs).

### Antibodies and Western blotting

An antibody recognizing the phosphorylated Rh2b CPD was raised against the phosphorylated Rh2b CPD peptide NNDHL(pS)NYADKE (3227–3239 aa) according to the manufacturer's protocols (Eurogentec). The antibody was purified in two steps. First, a column containing the phosphorylated peptide was used and all peptide antibodies were captured. In a second step, the specificity of mixed antibodies was improved by use of a column containing the unphosphorylated peptide with subsequent clearance of the flow through of non-specific IgG antibodies.

For immunoblots, parasite proteins from a synchronized culture were separated on 7.5% (Bio-Rad Laboratories) or 10% SDS/PAGE and transferred on to nitrocellulose membranes (Schleicher & Schuell). Membranes were blocked with 6% skimmed milk and incubated in the first antibody overnight. Monoclonal anti-HA or monoclonal anti-GFP (green fluorescent protein) were used at a 1:1000 dilution (Roche), phospho-specific anti-pRh2b antibody was used at a 1:3000 dilution, polyclonal anti-BiP (immunoglobulin heavy-chain-binding protein) was used at a 1:2000 dilution [28] and polyclonal anti-GST antibody (Genscript) was used at a 1:2000 dilution. The secondary antibodies used were HRP (horseradish peroxidase)-conjugated donkey anti-(rabbit IgG) (1:3000 dilution) or HRP-conjugated goat anti-(rat IgG) (1:3000 dilution) or HRP-conjugated goat anti-(mouse IgG) (1:3000 dilution) (Dianova). The immunoblots were developed by chemiluminescence using ECL (enhanced chemiluminescence; GE Healthcare).

### Immunoprecipitation and Lambda phosphatase treatment

A total of 120 ml of 8% synchronized Rh2b–HA parasites were harvested in the late schizont stage and the parasites were released from the host cells using saponin. Pure parasites were washed in ice-cold PBS and lysed in 50 mM Tris/HCl (pH 7.5), 150 mM NaCl, 0.5% NP-40 (Nonidet P40), Complete™ Protease inhibitor and Phosphatase Inhibitor Cocktail I. After separation of the membrane fraction by centrifugation, the supernatant was mixed with an anti-HA antibody (Roche) to a final concentration of 1 μg/ml and incubated with rolling at 4°C for 2 h. Protein G–agarose beads (50 μl; Genscript) were added and rolling continued overnight. Beads were then pelleted by centrifugation and washed three times in lysis buffer with two additional wash steps in lysis buffer without detergent. For phosphatase treatment, beads were split and one part was mixed with 400 units of Lambda PMP (protein metallophosphatase; New England Biolabs), whereas the other served as a control. Both were incubated for 15 min by 30°C, mixed with 5× SDS loading dye, boiled for 3 min at 95°C and separated by SDS/PAGE (7.5% gel) to perform Western blotting. For immunoprecipitation of CK2–HA, 40 ml of 8% mixed-stage parasites were released from the host cells by saponin and lysed as indicated above. After separating the soluble and the membrane fraction, the soluble lysate was pre-incubated with Protein G beads (Genscript) for 2 h, incubated with a final concentration of 1.5 μg/ml monoclonal anti-HA antibody for 3 h and finally the antigen–antibody complex was bound to Protein G beads overnight. After washing the antigen–antibody bead complex seven times in lysis buffer, beads were stored until use at −80°C. For the phosphorylation assays a volume of 5 μl of beads was used.

### Imaging and IFAs (immunofluorescence assays)

IFAs of Rh2b–HA parasites were performed on fixed parasites using ice-cold acetone for 30 min. After rehydration [10 min PBS at room temperature (22°C)] parasites were incubated with anti-HA (1:500 dilution) antibody solution, washed and incubated in secondary goat anti-rat antibody coupled to Alexa Fluor™ 594 (1:2000 dilution) (Molecular Probes) together with DAPI (4′,6-diamidino-2-phenylindole; 1 μg/ml; Roche). Parasites expressing CK2–HA were fixed using glutaraldehyde/formaldehyde [29], incubated with anti-HA antibody (1:500 dilution), washed and incubated with secondary goat anti-rat antibody coupled to Alexa Fluor™ 488 (1:2000 dilution; Molecular Probes) together with 1 μg/ml DAPI. All antibody dilutions were prepared in 3% BSA. Immunofluorescence and live cell images were observed and captured using a Zeiss Axioskop 2plus microscope with a Hamamatsu Digital camera (Model C4742-95). Single images were processed in Adobe Photoshop CS4.

## RESULTS

### Rh2b is phosphorylated *in vitro* and phosphorylation is PKA-independent

**Figure 1:**
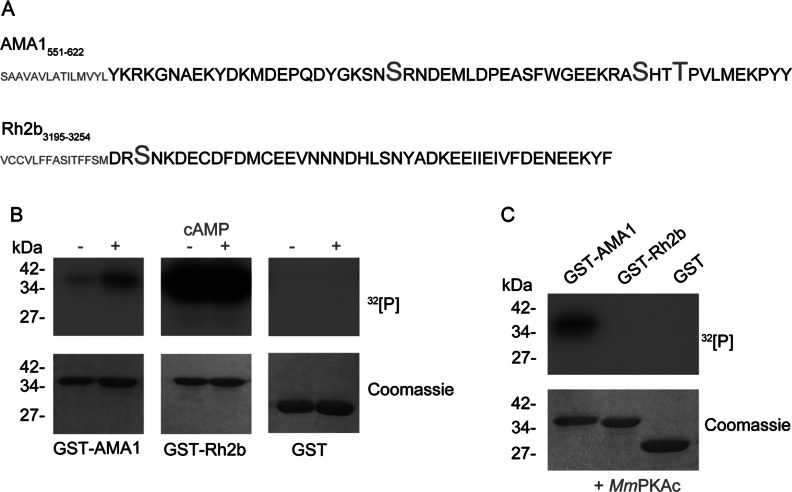
*In vitro* phosphorylation of Rh2b by parasite lysate (**A**) Primary sequence of the CPD of AMA1 and Rh2b. Amino acids predicted to be phosphorylated are shown in large font. Partial sequence of the transmembrane domain is depicted in small font. (**B**) *In vitro* radioactive [γ-^32^P]ATP phosphorylation assays of recombinant GST–AMA1 and GST–Rh2b fusion proteins in the presence (+) or absence (−) of 10 μM cAMP using schizont parasite lysate. Purified GST was used as a control. The loading of recombinant proteins is shown in the Coomassie Blue-stained SDS/PAGE. (**C**) *In vitro* radioactive [γ-^32^P]ATP phosphorylation assays of recombinant GST–AMA1, GST–Rh2b and GST using recombinant *Mm*PKAc. The molecular mass is shown in kDa on the left-hand side of the blots.

Recombinant cytoplasmic domains of Rh2b and AMA1 (Figure 1A) were used in *in vitro* phosphorylation assays with late-stage parasite lysate in the presence or absence of 10 μM cAMP. AMA1 was included as a control substrate. Whereas phosphorylation of recombinant GST–AMA1 was stimulated by cAMP, GST–Rh2b was already strongly phosphorylated in the absence of external cAMP. GST alone was not phosphorylated (Figure 1B). To underline the PKA-independent phosphorylation of Rh2b, the GST-fusion proteins were incubated with recombinant *Mm*PKAc. Although *Mm*PKAc phosphorylated AMA1–GST, it showed no reactivity against GST–Rh2b or the GST tag alone (Figure 1C). These findings suggested that Rh2b is not a substrate of PKA, but is the target of other kinases present in late-stage parasite material.

### Ser^3233^, but not Ser^3213^, is essential for *in vitro* phosphorylation of Rh2b

**Figure 2:**
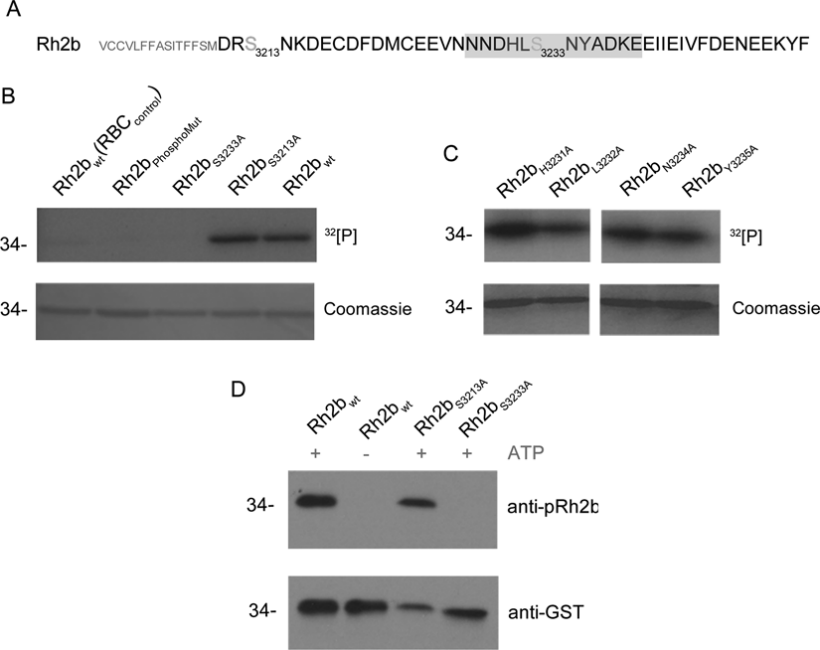
*In vitro* phosphorylation of the Rh2b mutants (**A**) Targeted amino acids in the mutational analysis are the numbered serine residues and those adjacent to Ser^3233^ (HL and NY). The primary sequence of the Ser^3233^-phosphorylated synthetic peptide used for raising an antibody (anti-pRh2b) is shaded in grey. (**B**) *In vitro* radioactive [γ-^32^P]ATP phosphorylation of recombinant Rh2b CPD serine mutants. Either both (Rh2b_PhosphoMut_) or a single serine residue were substituted with alanine. Wild-type Rh2b incubated with uninfected RBC lysate [Rh2b_wt_(RBC_control_)] or with schizont lysate (Rh2b_wt_) are used as a negative or positive control respectively. (**C**) *In vitro* radioactive [γ-^32^P]ATP phosphorylation of additional point mutants targeting Ser^3233^ adjacent amino acids. Upper panel, autoradiography. Lower panel, Coomassie-stained SDS/PAGE. (**D**) Western blot analysis of recombinant wild-type and mutant Rh2b CPD that was subjected to *in vitro* phosphorylation in the absence or presence of ATP prior to SDS/PAGE. Western blot analysis of these proteins reveal that the anti-pRh2b antibody exclusively recognizes phosphorylated Rh2b CPD at Ser^3233^ (upper panel). Anti-GST antibody was used as a loading control (lower panel). The molecular mass is shown in kDa on the left-hand side of the blots.

The CPD of Rh2b encodes four putative phospho-acceptor sites (Ser^3213^, Ser^3233^, Tyr^3235^ and Tyr^3253^), but only two are predicted to be phosphorylated by NetPhos 2.0 (http://www.cbs.dtu.dk/services/NetPhos/; Figure 2A, Tyr^3253^ score 0.968 and Ser^3213^ score 0.961) [30]. Owing to the absence of tyrosine kinases in the genome of the parasite and the low abundance of peptides phosphorylated on tyrosine residues recovered in proteomic approaches [31,32], only the predicted Ser^3213^, as well as Ser^3233^ as an additional putative phospho-acceptor site, were targeted for further analyses.

Recombinant Rh2b with either both or a single serine residue substituted with alanine were used in *in vitro* phosphorylation assays (Figure 2B). The substitution of the two serines (Ser^3213^ and Ser^3233^) resulted in a protein (Rh2b_PhosphoMut_) that is not susceptible for *in vitro* phosphorylation with parasite lysate, indicating that one or both of these residues are indeed targets for phosphorylation. Using single point mutations, Ser^3233^, but not the predicted Ser^3213^, was identified as the essential residue for this modification *in vitro* (Figure 2B). In control experiments, using extracts from uninfected erythrocytes, no significant phosphorylation was observed under the experimental conditions used, suggesting that erythrocyte kinases are not involved [Rh2b_wt_(RBC_control_), Figure 2B]. To verify Ser^3233^ as a genuine phospho-acceptor site *in vitro*, point mutations substituting adjacent residues including Tyr^3235^ were generated (Figure 2A, HL and NY). Efficient [γ^32^]phosphate transfer on these mutant Rh2b proteins supported the role of Ser^3233^ for phosphorylation (Figure 2C).

Finally, a phospho-specific Ser^3233^ anti-Rh2b antibody was raised against a phosphorylated peptide (Asn^3228^–Glu^3239^; Figure 2A, shaded in grey) and validated using wild-type and mutant GST–Rh2b proteins that were pre-incubated with parasite lysate in the presence or absence of the phosphate donor ATP. Western blot analysis using either the phospho-specific antibody (anti-phospho-Rh2b) or anti-GST antibody (as a loading control) confirmed Ser^3233^ phosphorylation and the phosphorylation specificity of the anti-phospho-Rh2b antibody (Figure 2D).

### Rh2b CPD as a potential *Pf*CK2 (*P. falciparum* CK2) substrate

**Figure 3:**
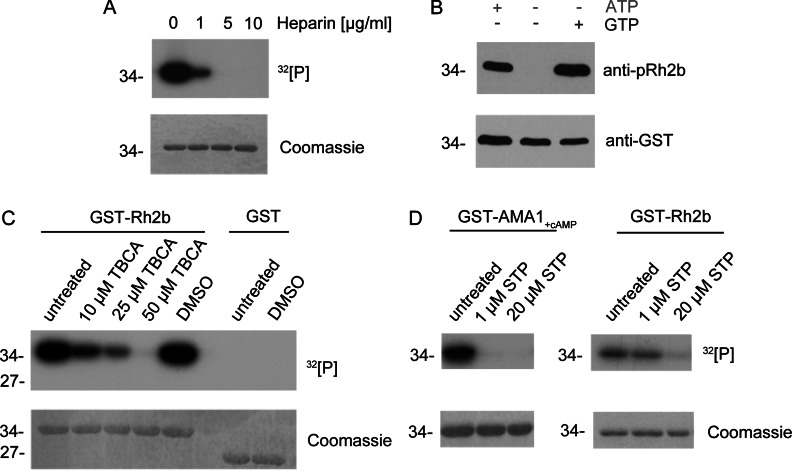
Inhibition of *in vitro* phosphorylation (**A**) Recombinant Rh2b was used in *in vitro* phosphorylation assays in the presence of [γ-^32^P]ATP with parasite lysate and different concentrations of the known CK2 inhibitor heparin. Phosphorylation as reduced by 1 μg/ml and eliminated by 5 and 10 μg/ml heparin. Upper panel, autoradiography. Lower panel, Coomassie Blue-stained SDS/PAGE. (**B**) The kinase, mediating Rh2b phosphorylation, was able to use ATP as well as GTP as phosphate donors. Rh2b CPD was not phosphorylated by parasite lysate in the absence of a phosphate donor. Upper panel, detection of phosphorylated Rh2b using the phospho-specific anti-pRh2b antibody. Lower panel, anti-GST antibody was used as a loading control. (**C**) The known CK2-specific inhibitor TBCA inhibited Rh2b CPD phosphorylation in a dose-dependent manner. In presence of parasite lysate, 10 μM TBCA reduced Rh2b phosphorylation, whereas 50 μM TBCA entirely eliminated phosphorylation. The solvent (DMSO) had no effect on Rh2b phosphorylation. Upper panel, autoradiography. Lower panel, Coomassie Blue-stained SDS/PAGE. (**D**) Phosphorylation of Rh2b CPD by parasite lysate was resistant to staurosporine (STP) treatment. Whereas cAMP-dependent AMA1 phosphorylation was inhibited by 1 μM staurosporine, Rh2b phosphorylation was resistant and only affected by higher doses. Upper panel, autoradiography. Lower panel, Coomassie Blue-stained SDS/PAGE. The molecular mass is shown in kDa on the left-hand side of the blots.

The kinase prediction tool NetPhosK 1.0 [33] predicts the identified phosphorylation site Ser^3233^ of Rh2b CPD to be a substrate of either CK2 or PKC (protein kinase C). Although the role of CK2 in blood-stage parasite proliferation is well established [25,34], no *bona fide* homologue for PKC could be identified in the *Plasmodium* genome [35], thus implicating CK2. CK2 features several characteristics that allow a clear distinction from other kinases. First, it is sensitive to low amounts of heparin [36] and secondly, it is able to use GTP as well as ATP as a phosphate donor [37]. In order to address the putative involvement of CK2 in Rh2b phosphorylation, increasing concentrations of heparin were used in *in vitro* phosphorylation assays with parasite lysate. This polyanionic compound is known to inhibit CK2 of several organisms [38]. Owing to its acidophilic preferences, the CK2 α subunit is especially targeted by heparin. Specific inhibition is reported in the range of 0.1–20 μg/ml [24,39–41]. The phosphorylation of Rh2b CPD was markedly reduced in the presence of 1 μg/ml (0.16 IU) heparin (Figure 3A). Higher amounts [5 and 10 μg/ml (0.8 and 1.6 IU)] completely abolished phosphorylation (Figure 3A). In contrast, a heparin concentration of 10 μg/ml did not markedly interfere with cAMP-dependent phosphorylation of the AMA1 CPD (Supplementary Figure S1 at http://www.biochemj.org/bj/452/bj4520457add.htm).

To pursue CK2 as a potential kinase involved in the phosphorylation of Rh2b, the co-substrate specificity of the kinase reaction was analysed. Most protein kinases use ATP as a phosphate donor, whereas very few, including *Pf*CK2 [34], are able to use GTP [42–44]. To test if Rh2b phosphorylation can occur in the presence of GTP, recombinant GST–Rh2b was incubated with parasite lysate in the presence of ATP or Mn^2+^ GTP and analysed for its phosphorylation status. Rh2b was found to be phosphorylated in the presence of ATP as well as in the presence of GTP, but not in the absence of a phosphate donor (Figure 3B).

Having established two hallmarks of CK2 phosphorylation (heparin sensitivity and phosphorylation in the presence of GTP as the phosphate donor) a CK2-specific inhibitor, TBCA [45], was used in *in vitro* phosphorylation assays. TBCA is a highly specific inhibitor of CK2 with a reported IC_50_ value of 0.11 μM for the purified rat and human enzymes. Its specificity was tested with 30 kinases where it most strongly inhibited rat CK2. Additionally, in the lysates of TBCA-treated Jurkat cells, CK2 activity was significantly reduced by concentrations of 5–50 μM [45].

Rh2b phosphorylation by parasite lysate was significantly reduced by a TBCA concentration of 10 μM, whereas 50 μM TBCA completely abolished its modification (Figure 3C). These assays were complemented by the use of the broad ATP-competitive kinase inhibitor staurosporine (Figure 3D). This inhibitor affects a variety of kinases in the nanomolar range, but fails to bind efficiently the small active cleft of the CK2 catalytic domain because of its large molecular size [46]. Purified CK2 α subunit from rats is reported to have a staurosporine IC_50_ value of 19 μM, whereas the enzyme's autophosphorylation is not affected by even a 32 μM concentration [47]. A previous study showed that *T. gondii* CK2-dependent phosphorylation is resistant to a 10 μM concentration of staurosporine using a *Toxoplasma* cell lysate [24].

*In vitro* Rh2b phosphorylation was not affected by 1 μM staurosporine, although cAMP-dependent AMA1 phosphorylation was efficiently blocked (Figure 3D). A higher concentration of staurosporine (20 μM) affected GST–Rh2b phosphorylation, which might suggest a different sensitivity to staurosporine as already described for its *T. gondii* homologue.

**Figure 4:**
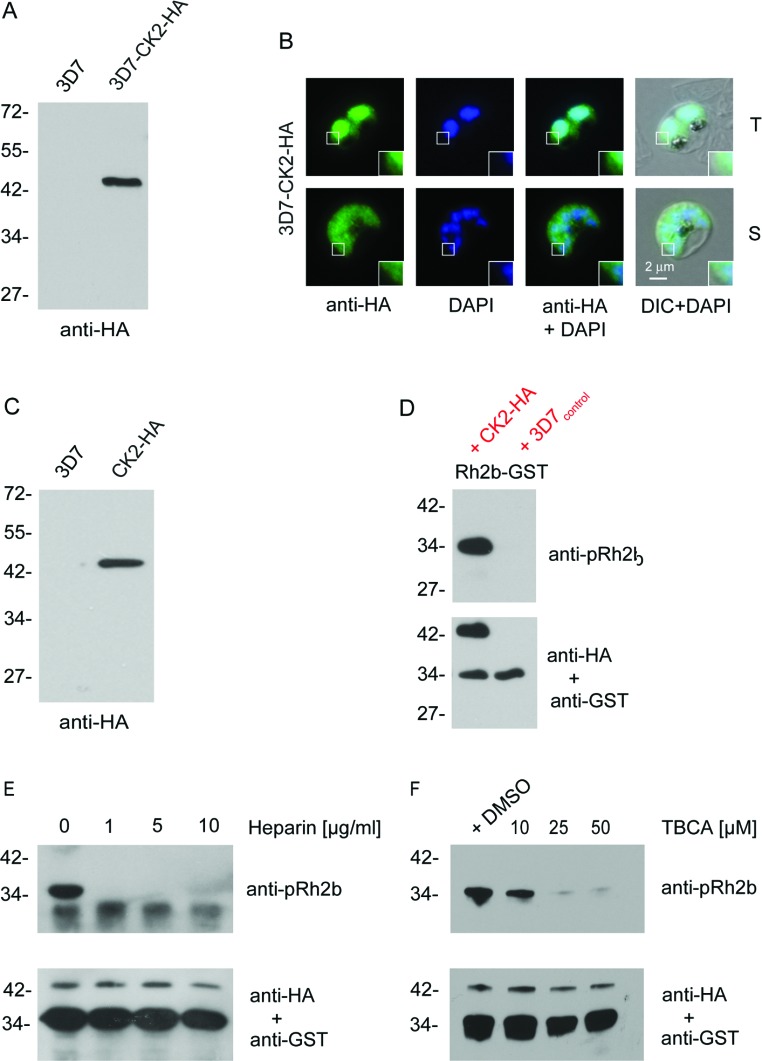
Expression, purification of *Pf*CK2 α and Rh2b *in vitro* phosphorylation (**A**) Western blot analysis of transgenic CK2–HA parasites that express an endogenous (3D7–CK2–HA) fusion protein. Expression of the fusion proteins was confirmed using an anti-HA antibody. The parental 3D7 strain (3D7) was used as a control. (**B**) Immunofluorescence microscopy using endogenously derived *Pf*CK2α–HA. Localization with the anti-HA antibodies (green) confirmed expression in the nucleus (blue, DAPI) and cytosol of fixed parasites in the trophozoite (T) and schizont (S) stage. Scale bar indicates 2 μm. Enlargements of selected areas are marked with a white square. DIC, differential interference contrast microscopy. (**C**) Western blot analysis of transgenic CK2–HA parasites that overexpress CK2–HA. Expression of the fusion proteins was confirmed using an anti-HA antibody. This cell line was used to immunopurify CK2–HA for subsequent phosphorylation assays. (**D**) Western blot analysis of phosphorylated Rh2b CPD using immunoprecipitated CK2–HA. Phosphorylation was detected using the phospho-specific anti-pRh2b antibody. CK2 was visualized with an anti-HA antibody and an anti-GST antibody was used as the loading control. (**E**) Western blot analysis of CK2-dependent phosphorylation of Rh2b in the presence of different heparin concentrations. CK2 was visualized with an anti-HA antibody and an anti-GST antibody was used as the loading control. (**F**) Western blot analysis of CK2-dependent phosphorylation of Rh2b CPD in the presence of different TBCA concentrations. CK2 was visualized with an anti-HA antibody and an anti-GST antibody was used as the loading control. The molecular mass is shown in kDa on the left-hand side of the blots.

To further test the involvement of CK2 in Rh2b phosphorylation, the CK2 α subunit-encoding gene (PF3D7_1108400) was either endogenously HA-tagged (3D7–CK2–HA, Figure 4B and Supplementary Figures S2A and S2B at http://www.biochemj.org/bj/452/bj4520457add.htm) or episomally overexpressed (CK2–HA) in 3D7 parasites (Figure 4C and Supplementary Figures S2C and S2D). Although the anti-HA antibody detected in both cell lines a protein band of ~42 kDa, it failed to detect a protein in the parental 3D7 cell line. As described previously [25] the endogenously tagged protein was localized in the parasite's cytosol, as well as in the nucleus of trophozoite stage parasites. In the schizont stage of the parasite the dual localization of endogenously tagged CK2 was less pronounced and appeared predominantly cytosolic (Figure 4B).

Immunoprecipitated HA-tagged CK2 purified from CK2–HAoverexpressing parasites (Supplementary Figure S3 at http://www.biochemj.org/bj/452/bj4520457add.htm) phosphorylated Rh2b at Ser^3233^ (Figure 4D), and this phosphorylation was sensitive to heparin treatment (Figure 4E) and to increasing amounts of TBCA (Figure 4F), thus showing similar properties to the assay with total parasite extracts (Figures 3A and 3C). Finally, the recombinant catalytic subunit of *Zm*CK2α (shown to share 65% identity with *Pf*CK2 α [34]) was utilized to examine its ability to phosphorylate GST–AMA1, GST–Rh2b and the GST tag alone (Supplementary Figure S3). Although the enzyme was able to phosphorylate GST–Rh2b, no phosphorylation was detected for GST–AMA1 as well as GST itself. An additional phosphorylation band detected at 42 kDa suggests autophosphorylation of *Zm*CK2α and showed the use of equal amounts of the kinase in the assays.

### Rh2b is phosphorylated late in mature schizonts

**Figure 5:**
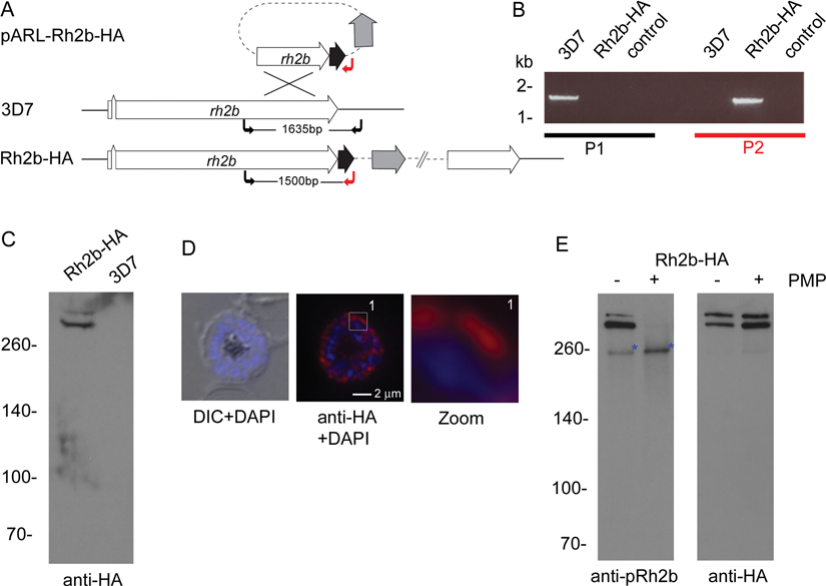
*In vivo* phosphorylation of Ser^3233^ in schizonts (**A**) Schematic drawing of the Rh2b–HA 3′ replacement approach in 3D7 parasites. The *rh2b* gene has a two exon structure and an open reading frame of 9765 bp. Approximately 1 kb of the 3′-end was fused with the coding sequence of a triple HA epitope (black) and cloned into a pARL derivate (pARL-3′Rh2b-HA). The hDHFR (grey box) of the plasmid allowed the selection of transgenic parasites. The position of the oligonucleotides used for diagnostic PCR are shown with black and red arrows. (**B**) Integration of the plasmid into the *rh2b* locus was confirmed by PCR using two different primer sets (black and red) on gDNA of 3D7 and Rh2b–HA parasites. (**C**) Expression of Rh2b–HA was verified by Western blot analysis using anti-HA specific antibodies. Using schizont parasite material two proteins larger than 260 kDa are detectable exclusively in the transgenic parasite line Rh2b–HA. The size of the proteins correspond to the full-length (M_W_=385 kDa) and a N-terminally processed form of Rh2b. (**D**) Expression was also verified by IFA using anti-HA antibodies. Scale bar, 2 μm. DIC, differential interference contrast microscopy. (**E**) *In vivo* phosphorylation of Rh2b was investigated by the Ser^3233^ phospho-specific antibody (anti-pRh2b) using immunoprecipitated Rh2b–HA. The phospho-specificity of the antibody was verified by the use of Lambda PMP. Prior to Western blot analysis the immunoprecipitated material was either incubated with (+) and without (−) phosphatase and subjected to anti-pRh2b detection. The Western blots were re-probed with the anti-HA antibody. Whereas the anti-HA antibody detects two forms of Rh2b independently of phosphatase treatment, the phosphorylation-specific antibody detects Rh2b CPD phosphorylation predominantly in the lower mass protein. PMP treatment abolishes anti-pRh2b recognition of Rh2b–HA. A putative cross-reactive protein is marked with a blue asterisk. The molecular mass in shown in kDa on the left-hand side of the blots.

To facilitate the investigation of Rh2b phosphorylation *in vivo*, a transgenic parasite line was generated expressing endogenous Rh2b with a C-terminal 3×HA tag (Figure 5A). Single-cross-over integration of the plasmid into the *rh2b* locus was confirmed by PCR (Figure 5B) and expression of HA-tagged Rh2b was verified using an anti-HA antibody on schizont stage parasites. As reported previously [15,48], Rh2b–HA can be detected as a presumably full length (>300 kDa) and N-terminally processed protein with a molecular mass of about 300 kDa (Figure 5C) and is localized at the apical pole of nascent merozoites (Figure 5D).

To analyse *in vivo* phosphorylation of Rh2b CPD, Rh2b–HA was immunoprecipitated with an anti-HA antibody. Precipitated proteins were incubated with and without Lambda PMP prior to Western blot analysis using the anti-phosphoSer^3233^ antibody. This antibody (like the anti-HA antibody) recognized two protein bands at 300 kDa and above (Figure 5E) and protein detection was sensitive to phosphatase treatment. An additional 260 kDa band, insensitive to phosphatase treatment, was also detected by this antibody that might represent an unrelated cross-reactive protein.

Whereas the anti-HA antibody detected both Rh2b forms with approximately the same intensity, notably, the lower migrating band (resembling the N-terminally processed Rh2b) was the dominant form detected by the anti-pRh2b antibody, suggesting that the full-length Rh2b protein was significantly less phosphorylated (Figure 5E).

The specificity of the anti-Rh2b antibody and Rh2b CPD phosphorylation *in vivo* was confirmed further by episomal overexpression of the two variants of the CPD as GFP-fusion proteins that allowed the differential targeting of the CPD to two specific membranes: the plasma and rhoptry membranes. In both membrane systems the CPD of Rh2b were phosphorylated in the late stages (Supplementary Figure S4 at http://www.biochemj.org/bj/452/bj4520457add.htm).

## DISCUSSION

Previous studies on the CPD of AMA1 revealed specific phosphorylation mediated by PKA as one essential step during the invasion process [3,22]. However, it remains unclear if this post-translational modification is unique to AMA1 and its function, or if it is shared between CPDs of other adhesins, like members of the EBA or Rh family. These domains show no overall homology, conserved amino acid pattern or phosphorylation sites. Although publications analysing protein phosphorylation on a global scale highlight the importance of this post-translational modification in all physiological processes in the asexual proliferation of the parasite [31,32], no phosphorylated peptides were identified matching the CPD of any of these type I adhesins except for AMA1. This could be explained by low abundance, under-representation of transmembrane proteins or the biophysical properties of the phosphopeptide that might impede their identification by shotgun MS approaches. However, it might simply also reflect their *in vivo* absence. By contrast, phosphorylation prediction tools like NetPhos 2.0 do predict at least one phosphorylation site (above the arbitrarily implemented threshold of 0.5) within the analysed CPDs except for EBA175 and Rh1 (Supplementary Figure S5 http://www.biochemj.org/bj/452/bj4520457add.htm).

In the present study we show that the CPD of Rh2b is phosphorylated at a single residue (Ser^3233^) in a cAMP-independent manner in *in vitro* assays. Its phosphorylation appears to correlate *in vivo* in late schizont stages mainly with N-terminal processing. A substantial line of evidence implicates *Pf*CK2 in Rh2b phosphorylation: Ser^3233^ phosphorylation is sensitive to low amounts of heparin [36] and to TBCA treatment, a specific CK2 inhibitor [45]. Furthermore phosphorylation occurs in the presence of the phosphate donors ATP as well as GTP, an ambivalence well established for CK2-catalysed phosphorylation [34,37]. Finally, immunoprecipitated *Pf*CK2 as well as recombinant *Zm*CK2α phosphorylates Ser^3233^. CK2 is a highly conserved kinase that can be found ubiquitously in eukaryotic organisms [49]. Most eukaryotic CK2 holoenzymes consist of two α subunits and two regulatory β subunits, whereas the malaria parasite expresses two regulatory subunits β1 and β2 that appear to be essential for parasite development [25,34]. In contrast with the established invasion related kinases such as PKA [22] and CDPK1 [23] that are transcriptionally expressed at the late schizont stage, all three CK2 genes (α, β1 and β2) are not transcriptionally up-regulated in the schizont stage [50]. However, CK2 is present in the schizont stage (Figure 4B), and all three subunits are recognized as an integral part of the schizont/merozoite proteome [25,31,51]. Investigation of *in vivo* phosphorylation of *Pf*GAP45 (*P. falciparum* gliding-associated protein 45) implicated CK2 activity in staurosporine-resistant phosphorylation [52] and data from immunoprecipitation experiments found an interaction between the CK2 β1 subunit and rhoptry-resident proteins [25].

The CK2-dependent phosphorylation of the CPD of Rh2b at Ser^3233^ might reveal another molecular target of this kinase. Future work has to uncover the physiological role of this phosphorylation. Our preliminary results using mutagenic parasites expressing the S3233A mutant form of Rh2b suggest that Rh2b CPD phosphorylation may not be directly linked to the invasion process (Supplementary Figure S6 at http://www.biochemj.org/bj/452/bj4520457add.htm). We found that the mutation S3233A in the CPD of a chimaeric Rh2a/b invasion ligand has no significant impact on the use of invasion pathways by D10 parasites (Supplementary Figure S6). We therefore propose that phosphorylation of Ser^3233^ might trigger a pre-invasive process that could be connected to translocation of the Rh2b protein or to a putative role in signalling events in merozoites primed for invasion.

## Online data

Supplementary data
