# Supplementary material for: Specific phosphorylation of the PfRh2b invasion ligand of Plasmodium falciparum
Source: Biochem J. 2013 May 31;452(Pt 3):457–66. doi: 10.1042/BJ20121694 (PMC3671792; doi:10.1042/BJ20121694)
Supplement: Supplementary data [file bj4520457add.pdf]

BJ Disease

Biochem. J. (2013) **452**, 457–466 (Printed in Great Britain) doi:10.1042/BJ20121694

SUPPLEMENTARY ONLINE DATA

Specific phosphorylation of the *Pf*Rh2b invasion ligand of *Plasmodium falciparum*

Klemens ENGELBERG*, Aditya S. PAUL†, Boris PRINZ*, Maya KONO*, Wilhelm CHING‡, Dorothee HEINCKE*, Thomas DOBNER§, Tobias SPIELMANN*, Manoj T. DURAISINGH† and Tim-Wolf GILBERGER*§[1]

*Bernhard-Nocht-Institute for Tropical Medicine, Department of Molecular Parasitology, Hamburg, Germany, †Harvard School of Public Health, Department of Immunology and Infectious Diseases, Boston, U.S.A., ‡Heinrich Pette Institute, Leibniz Institute for Experimental Virology, Department of Molecular Virology, Hamburg, Germany, and §M.G. DeGroote Institute for Infectious Disease Research and Department of Pathology and Molecular Medicine, McMaster University, Hamilton, Canada

MATERIALS AND METHODS

Parasite strains, transfection and single-cross-over integration

D10 transfections expressing the mutant Rh2b CPD were produced as described previously [1]. Single cross-over transgenic parasite clones were selected by a limiting dilution. D10 parasites expressing the wild-type Rh2a or Rh2b CPD in place of the native domain at the *rh2a* chromosomal locus (2a-wt and 2b-wt) were previously generated in D10 parasites [1]. This replacement of the endogenous Rh2a cytoplasmic domain by the Rh2b domain stimulates use of the inactive invasion ligand (Rh2a) through a pathway characteristic of Rh2b. This system thus provides a sensitive mean to monitor the gain of function conferred by transgenic CPDs [1].

Erythrocyte invasion assays

3D7 parasites as well as the D10 2b-wt cell line invade well into cells treated with a combination of neuraminidase and trypsin, whereas parasites lacking any copy of the Rh2b CPD like the parental strain D10 or the 2a-wt cell line virtually do not invade these cells [1].

To analyse the invasion phenotype of transgenic D10-derived parasites harbouring the S3213A or S3233A single mutations, as well as parasites with a double serine substitution, with the Rh2b domain, enzyme-treated erythrocytes were used. Invasion assays were performed as described previously [1–3]. Parasitized 'donor' cells were synchronized at least once with sorbitol (5 %) within two cycles before the start of the assay. After extensive washing with incomplete (I)-RPMI (i.e. without albumax nor bicarbonate), ring stage donor cells were treated with 2 volumes of an enzyme mix [100 m-units/ml neuraminidase (Roche), 1.5 mg/ml chymotrypsin (Sigma) in I-RPMI and 1.5 mg/ml trypsin (Sigma) in I-RPMI]. Receptor cells were handled similarly with a combination of neuraminidase (final concentration 66.7 m-units/ml) and trypsin (final concentration 1 mg/ml), for 1 h at 37 °C with gentle rocking. Following extensive washing in I-RPMI lacking hypoxanthine, enzyme-treated cells were resuspended in low-hypoxanthine (2.5 mg/l) RPMI medium and mixed with receptor cells at a ratio of 1:1 in a 96-well plate to a final parasitaemia of ∼0.8 % with 2 % haematocrit. After ∼48 h, to allow one round of re-invasion, 0.2 volumes of [^{3}H]hypoxanthine (0.05 μCi/μl in low-hypoxanthine RPMI) were added to each sample. All samples were further incubated for ∼20–24 hours before harvest on to glass filter plates for liquid scintillation counting of radioactive parasite material. Between live culture and harvest of cells, at least one cycle of freeze–thaw was performed to lyse the red blood cells.

Nucleic acids and constructs

D10 parasites expressing Rh2a/b S3233A (2b-S3233A, vector pAP26), Rh2a/b S3213A (2b-$S_3$213A, vector pAP25) and Rh2a/b S3233S3213A (2b-PM, vector pAP39) chimaera were generated by changing the appropriate wild-type codons in the 3′ replacement vector pJDD108 [1]. Successful integration in the D10 background was confirmed by Southern blotting analysis as described previously [1].

Rh2b fusion with either a rhoptry or plasma membrane targeting sequence was achieved by inserting the *rh2b*-encoding sequence of its CP (bp 9627–9762 of *rh2b*) in the previously published vectors pARL-20ARO_{WT}-GFP (rhoptry membrane) and pARL-20CDPK1-GFP [4] using the AvrII restriction site. Both vectors use the *ama1* promoter for late-stage restricted transcription of the transgene [5].

Phosphatase treatment

Phosphatase treatment of Rh2b–GFP_{Rhop} parasites was carried out as described previously [6] with late schizont material.

Time course experiments

To obtain highly synchronized parasites for subsequent time course experiments, parasites were treated as described previously [7]. Briefly, parasites were synchronized in the ring stage with 5 % D-sorbitol [8] and schizonts were harvested in the next cycle using a percoll (GE Healthcare) gradient. Schizonts were incubated in complete RPMI medium at 10 % haematocrit, rolling at 37 °C and reinvasion was allowed for 4 h. Parasites were treated again with 5 % D-sorbitol to remove schizont stage parasites and the time course was started after 32 h. Parasites were harvested every 4 h, lysed in saponin, washed with 1× PBS and pelleted parasites were resuspended in 5× SDS loading dye to preform subsequent Western blotting.

[1] To whom correspondence should be addressed (email tgilber@mcmaster.ca).

© 2013 The Author(s)
The author(s) has paid for this article to be freely available under the terms of the Creative Commons Attribution Licence (CC-BY) (http://creativecommons.org/licenses/by/3.0/) which permits unrestricted use, distribution and reproduction in any medium, provided the original work is properly cited.

Biochemical Journal www.biochemj.org

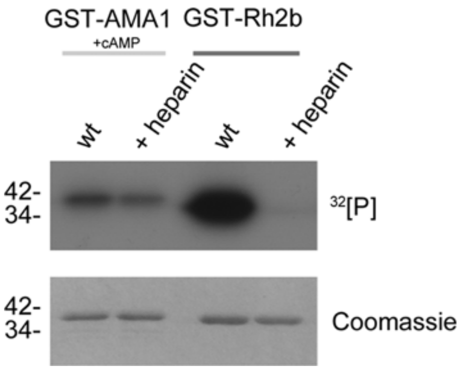

Figure S1 ***In vitro*** **phosphorylation of GST–AMA1 and GST–Rh2b in the presence of heparin**

GST–AMA1 and GST–Rh2b were phosphorylated in the presence of parasite lysate. The addition of cAMP stimulated the phosphorylation of GST–AMA1. Heparin was added at a concentration of 10 μg/ml. Upper panel, autoradiography. Lower panel, Coomassie Blue-stained SDS/PAGE. The molecular mass is shown in kDa on the left-hand side. wt, wild-type.

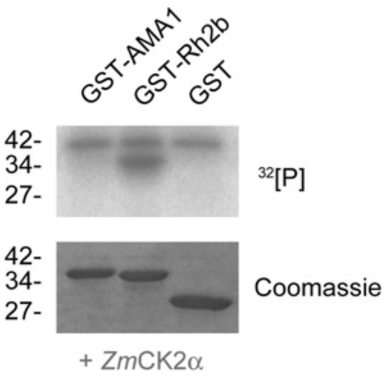

Figure S3 ***In vitro*** **phosphorylation of GST–Rh2b by *Zm*CK2α**

Recombinant *Zm*CK2α was used in *in vitro* phosphorylation assays in the presence of radioactive [γ-^{32}P]ATP. Substrates were recombinant GST–AMA1, GST–Rh2b and GST. Upper lane, ^{32}P incorporation was visualized using a PhosphoImager plate (FujiFilm) and a FLA-3000 luminometric detection system and was analysed by Image Gauge software. Lower lane, Coomassie Blue-stained SDS/PAGE. The molecular mass in shown in kDA on the left-hand side.

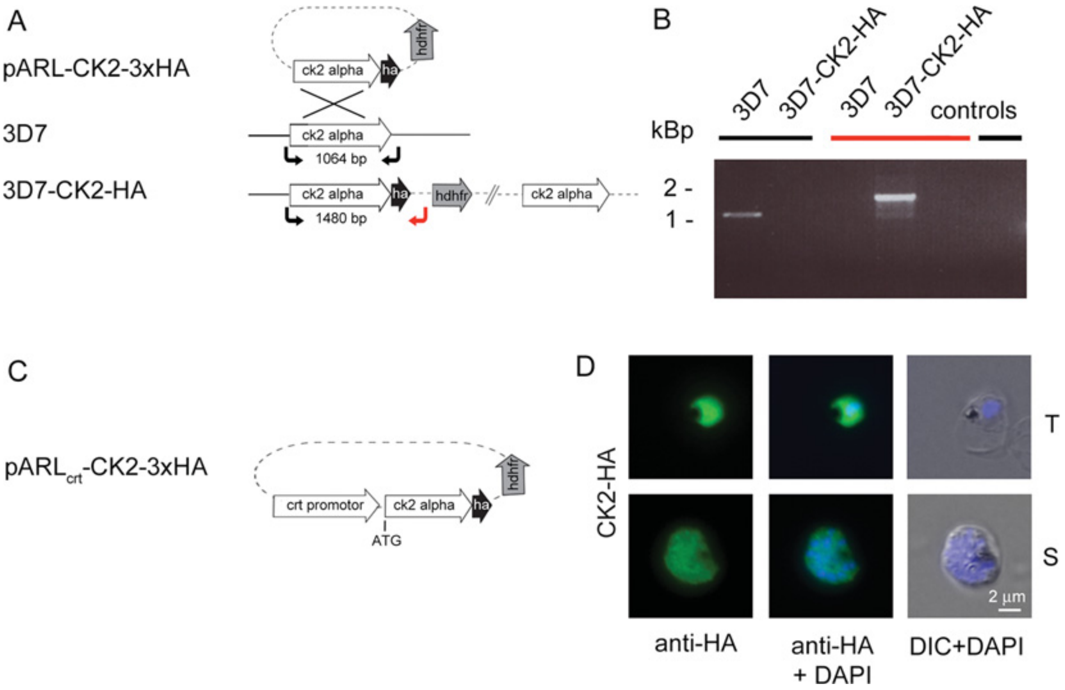

Figure S2 **HA tagging of the endogenous CK2 and localization of CK2–HA-overexpressing parasites**

(**A**) Schematic drawing of 3D7–CK2–HA 3′ replacement approach in 3D7 parasites. The pARL1a-ck2alpha3xha plasmid was used to replace the endogenous *ck2alpha* gen-locus in order to express CK2 as a HA-fusion protein. Selection of transgenic parasites expressing 3D7–CK2–HA was achieved by WR99210 and the selection marker hDHFR (grey box), encoded by the pARL plasmid. Black and red arrows indicate the position of oligonucleotides used for diagnostic PCR. The theoretical length of the amplified PCR products are indicated in bp. (**B**) Diagnostic PCR using parental 3D7 and transgenic 3D7–CK2–HA gDNA. PCR was carried out using either an oligonucleotide combination that detects the endogenous *ck2alpha* gen-locus (black) or oligonucleotides that detect the gen-locus after replacement with the pARL vector (red). As a control PCR (control) reactions without addition of gDNA were carried out. Fragment sizes are annotated in bp. (**C**) Schematic drawing of CK2–HA pARL1 plasmid to overexpress CK2 tagged with 3$\times$HA in the parasite. Expression was driven by the *crt* promoter. The start codon (ATG) of the *ck2alpha* open reading frame is indicated. (**D**) Localization of overexpressed *Pf*CK2 α subunit as a HA-fusion protein (CK2–HA) in 3D7 parasites. Transgenic parasites were fixed with glutaraldehyde/paraformaldehyde. The fusion protein was visualized in the trophozoite (T) and schizont (S) stage of the parasite by use of an anti-HA antibody (green). Parasites nuclei were stained with DAPI (blue). Scale bar indicates 2 μm. DIC, differential interference contrast microscopy.

© 2013 The Author(s)
The author(s) has paid for this article to be freely available under the terms of the Creative Commons Attribution Licence (CC-BY) (http://creativecommons.org/licenses/by/3.0/) which permits unrestricted use, distribution and reproduction in any medium, provided the original work is properly cited.

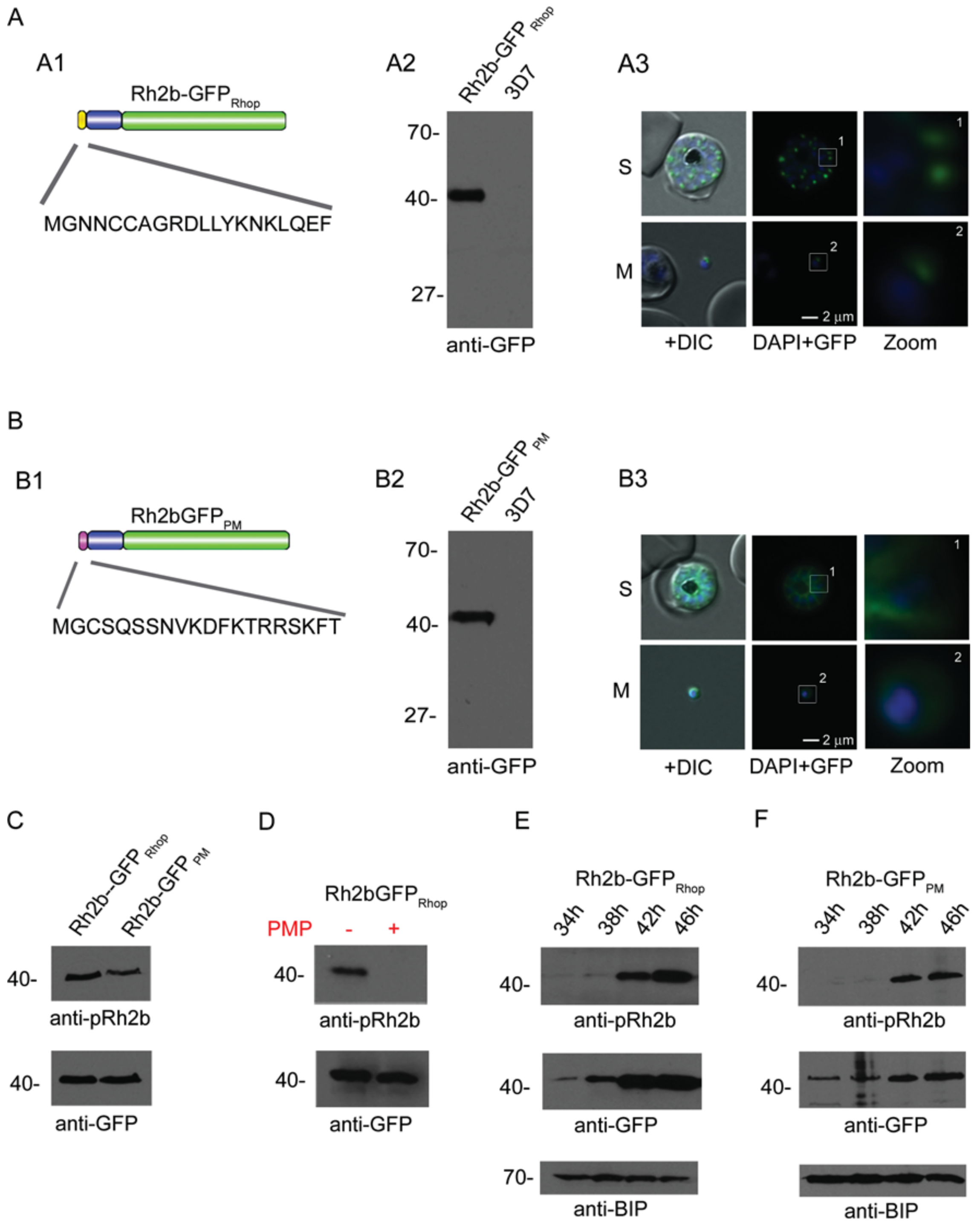

Figure S4 Spatiotemporal patterns of Rh2b phosphorylation

(**A** and **B**) Expression and localization of Rh2b as a GFP-fusion protein at the cytosolic face of the rhoptries (**A**) or the plasma membrane (**B**) using the N-termini (first 20 amino acids) of the rhoptry protein ARO (PlasmoDB accession number PF3D7_0414900) (A1) and the plasma membrane protein CDPK1 (PlasmoDB accession number PF3D7_0217500) (B1). The Rh2b CPD (blue) was cloned between the respective N-terminus and GFP (green bar). Expression at the schizont stage was controlled by the *ama*1 promoter and verified using Western blot analysis in combination with anti-GFP antibodies. In the transgenic, but not in the parental, parasite lines, a GFP-fusion protein can be detected at about 42 kDa using anti-GFP antibodies (M_W 35 kDa, A2 and B2). Using live-cell microscopy the fusion protein can either be detected at the apical tip (A3) or at the plasma membrane (B3) in nascent (S) or in free merozoites (M). Scale bar indicates 2 μm. DIC, differential interference contrast microscopy. (**C**) Phosphorylation of the Rh2b CPD, independent of its localization at the rhoptry or the plasma membrane, is detected by the phospho-specific anti-phospho-Rh2b antibody. (**D**) Detection of phosphorylated Rh2b CPD fusion proteins by anti-pRh2b antibody is sensitive to Lambda PMP treatment. (**E**) Time course experiments ($\pm$2 h window) using either rhoptry- or plasma membrane-derived Rh2b CPD reveals phosphorylation in the late stages of schizogony. Phosphorylation was detected by the anti-pRh2b antibody, expression of the fusion proteins was visualized using the anti-GFP antibody and the anti-BiP antibody was used to show equal loading of time points. Hours indicate the hours post merozoite invasion.

© 2013 The Author(s)
The author(s) has paid for this article to be freely available under the terms of the Creative Commons Attribution Licence (CC-BY) (http://creativecommons.org/licenses/by/3.0/) which permits unrestricted use, distribution and reproduction in any medium, provided the original work is properly cited.

AMA1
YKRKGNAEKYDKMDEPQDYGKSNSRNDEMLDPEASFWGEEKRASHTTPVLMEKPYY

MTRAP
RKEKTEKVVQEETKEENFEVMFNDDALKGKDNKAMDEEEFWALE

EBA140
RMGKSNEEYDIGESNIEATFEENNYLNKLSRIFNQEVQETNISDYSEYNYNEKNMY

EBA175
QAKYQSSEGVMNENNENNFLFEVTDNLDKLSNMFNQQVQETNINDFSEYHEDINDINFKK

EBA181
YRKNLDDEKGFYDSNLNDSAFEYNNNKYNKLPYMFDQQINVVNSDLYSEGIYDDTTTF

Rh1
SKNNKQEYDKEQEKQQQNDFVCDNNKMDDKSTQKYGRNQEEVMEIFFDNDYI

Rh2a
KTNSGDNNSNEINEAFEPNDDVLFKEKDEIIEITFNDNDSTI

Rh2b
DRSNKDECDFDMCEEVNNNDHLSNYADKEEIIEIVFDENEEKYF

Rh4
KNSNEPHHIFNIFQKEFSEADNAHSEEKEEYLPVYFDEVEDEVEDEVEDEDENENEVENENEDFNDI

Figure S5 Putative phosphorylation sites in invasion ligand CPDs

The cytoplasmic domains of AMA1 (PlasmoDB accession number PF3D7_1133400), MTRAP (PlasmoDB accession number PF3D7_1028700), EBA140 (PlasmoDB accession number PF3D7_1301600), EBA175 (PlasmoDB accession number PF3D7_0731500), EBA181 (PlasmoDB accession number PF3D7_0102500), Rh1 (PlasmoDB accession number PF3D7_0402300), Rh2a (PlasmoDB accession number PF3D7_1335400), Rh2b (PF3D7_1335300) and Rh4 (PlasmoDB accession number PF3D7_0424200) were analysed using NetPhos 2.0 [9] to predict putative serine and threonine phosphorylation sites (shown in large font) above a threshold of 0.5.

Table S1 Oligonucleotides used in the present study

List of all oligonucleotides used in the present study. Restriction sites are displayed in lower case and substitutions are underlined. UTR, untranslated region.

Primer	Sequence (5′→3′)
Rh2b sense BamHI	GCGCggatccGACAGATCAAATAAGGATGAATGCG
Rh2b antisense XhoI	GCGCctcgagATTAAAATATTTTTCTTCATTTTCATCAAACAC
Rh2b S3213>A sense BamHI	GCGCggatccGACAGAGCAAATAAGGATGAATGCG
Rh2b S3233>A sense	GAAGTAAATAATAATGATCACTTAGCGAATTATGCTGATAAAGAAG
Rh2b S3233>A antisense	CTTCTTTATCAGCATAATTCGCTAAGTGATCATTATTATTTACTTC
Rh2b H3231>A sense	GAAGTAAATAATAATGATGCCTTATCGAATTATGCTGATAAAGAAGAAATTATTG
Rh2b H3231>A antisense	CAATAATTTCTTCTTTATCAGCATAATTCGATAAGGCATCATTATTATTTAC
Rh2b L3232>A sense	GAAGAAGTAAATAATAATGATCACGCATCGAATTATGCTGATAAAGAAG
Rh2b L3232>A antisense	TTCTTCTTTATCAGCATAATTCGATGCGTGATCATTATTATTTACTTCTTCACAC
Rh2b N3234>A sense	GATCACTTATCGGCTTATGCTGATAAAGAAGAAATTATTGAAATTGTG
Rh2b N3234>A antisense	CTTCTTTATCAGCATAAGCCGATAAGTGATCATTATTATTTACTTCTTCACAC
Rh2b Y3235>A sense	GTAAATAATAATGATCACTTATCGAATGCTGCTGATAAAGAAG
Rh2b Y3235>A antisense	CTTCTTTATCAGCAGCATTCGATAAGTGATCATTATTATTTAC
Rh2b$_{\text{reporter}}$ sense AvrII	GCGCcctaggGACAGATCAAATAAGGATGAATGCG
Rh2b$_{\text{reporter}}$ antisense AvrII	GCGCcctaggAAAATATTTTTCTTCATTTTCATCAAACAC
Rh2b$_{\text{intecheck}}$ sense	CAAGAATTAGAAGAGC
Rh2b 3′ antisense	GATTTAATTATAGTGTTTTAAAGTTTAAAGC
Rh2b$_{\text{inte}}$ sense NotI	GCGCgcggccgcATTACACATCCTAGTAATATTGGAGG
oAP29 sense AvrII	AAAAAAcctaggGATCTTGGTGATGAAAAATTAATATTAAAAG
oAP33 antisense Acc65I	AAAAAAggtaccTTAAAAATATTTTTCTTCATTTTCATCAAAC
oAP40 sense	CTTATAGACAGAGCAAATAAGGATGAATG
oAP39 antisense	CATTCATCCTTATTTGCTCTGTCTATAAG
oAP42 sense	GATCACTTAGCAAATTATGCTG
oAP41 antisense	CAGCATAATTTGCTAAGTGATC
CK2α sense KpnI	GCGCggtaccATGTCGGTTAGCTCAATTAATAAAAAAATTTATATACC
CK2α antisense XbaI	GCGCtctagaTGATTCCTCACGGACTTCTC
CK2 3′ antisense	TTTGTTTGGAAAAACACC
CK2α_{inte} sense NotI	GAGAgcggccgcTCGGTTAGCTCAATTAATAAAAAAATTTATATACC
CK2α 5′ UTR	TTTTTAAAAAGAGACAGG
pGEX4T1 sense	CCAGCAAGTATATAGCATGG
pARL antisense	CAGTTATAAATACAATCAATTGG
ama1 sense	TCAAATTAATGTACTTGTTATAAATTGTACAAA
crt sense	AGTGAAAAGTTCTTCTCC

© 2013 The Author(s)
The author(s) has paid for this article to be freely available under the terms of the Creative Commons Attribution Licence (CC-BY) (http://creativecommons.org/licenses/by/3.0/) which permits unrestricted use, distribution and reproduction in any medium, provided the original work is properly cited.

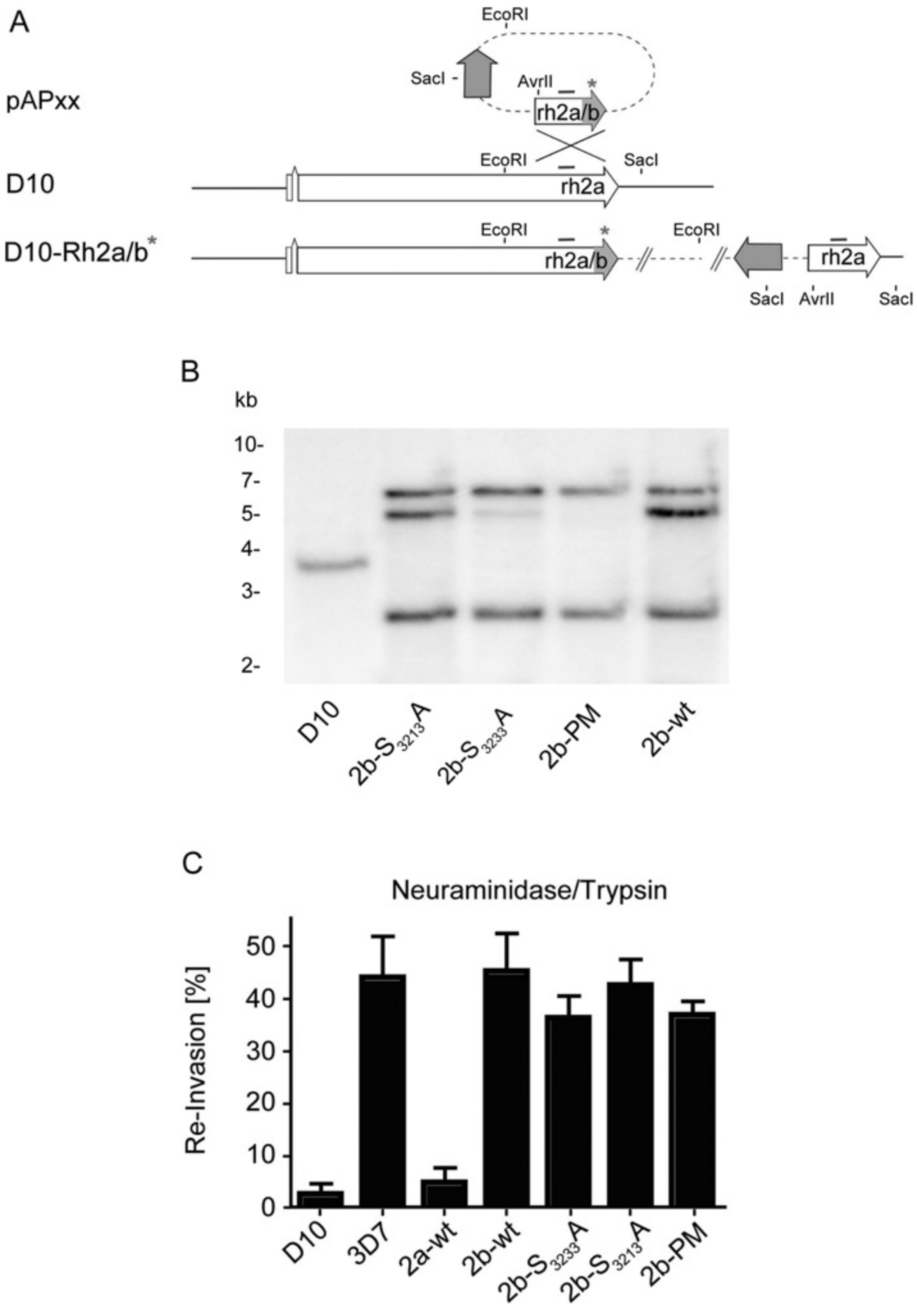

Figure S6 Invasion phenotype of the Rh2b S3233A parasite

(**A**) Schematic drawing of the Rh2b 3′ replacement approach in D10 parasites in order to express chimaeric Rh2a/b with Ser^{3213}, Ser^{3233} or both serines (2b-PM) substituted with an alanine residue. The following variants of chimaeric Rh2a/b were cloned into derivatives of pJDD108 [1]: 2b-S3213A, pAP25; 2b-S3233A, pAP26 and 2b-PM, pAP39. The mutant tail is indicated with an asterisk. Selection of transgenic parasites expressing these mutant Rh2a/b was done by WR99210 exploting the hDHFR (grey box) encoded by the plasmid. (**B**) Successful integration of transfection plasmids was verified by Southern blot analysis. Genomic DNA of transgene parasites was cut with the restriction enzymes AvrII, EcoRI and SacI and probed with a DNA fragment binding to the *rh2a* locus (shown in **A** as a bar above the protein). In D10 wild-type parasites this digestion resulted in a DNA fragment detected by the probe of ∼3750 bp. In transgenic cell lines, DNA fragments of ∼2640 bp and ∼6000 bp confirmed integration in the *rh2a* locus. An additional band at ∼5000 bp indicated episomal plasmid in these cell lines. (**C**) Re-invasion assays of the wild-type strains D10 and 3D7, as well as the transgenic strains 2a-wt, 2b-wt, 2b-S_{3233}A, 2b-S_{3213}A and 2b-PM into cells treated with a combination of neuraminidase and trypsin. The magnitude of invasion into these cells is expressed as a percentage value relative to fully-permitted invasion by the same strain (receptor cells not treated with enzymes). The indicated values are an average of two biological replicates. Each biological replicate was done in triplicate. The error bars are the 95 % confidence interval.

© 2013 The Author(s)
The author(s) has paid for this article to be freely available under the terms of the Creative Commons Attribution Licence (CC-BY) (http://creativecommons.org/licenses/by/3.0/) which permits unrestricted use, distribution and reproduction in any medium, provided the original work is properly cited.

REFERENCES

1 Dvorin, J. D., Bei, A. K., Coleman, B. I. and Duraisingh, M. T. (2010) Functional diversification between two related *Plasmodium falciparum* merozoite invasion ligands is determined by changes in the cytoplasmic domain. Mol. Microbiol. **75**, 990–1006

2 Fidock, D. A., Nomura, T. and Wellems, T. E. (1998) Cycloguanil and its parent compound proguanil demonstrate distinct activities against *Plasmodium falciparum* malaria parasites transformed with human dihydrofolate reductase. Mol. Pharmacol. **54**, 1140–1147

3 Reed, M. B., Caruana, S. R., Batchelor, A. H., Thompson, J. K., Crabb, B. S. and Cowman, A. F. (2000) Targeted disruption of an erythrocyte binding antigen in *Plasmodium falciparum* is associated with a switch toward a sialic acid-independent pathway of invasion. Proc. Natl. Acad. Sci. U.S.A. **97**, 7509–7514

4 Cabrera, A. L., Herrmann, S., Warszta, D., Santos, J. M., John Peter, A. T., Kono, M., Debrouver, S., Jacobs, T., Spielmann, T., Ungermann, C. et al. (2012) Dissection of minimal sequence requirements for rhoptry membrane targeting in the malaria parasite. Traffic **13**, 1335–1350

5 Treeck, M., Struck, N. S., Haase, S., Langer, C., Herrmann, S., Healer, J., Cowman, A. F. and Gilberger, T.-W. (2006) A conserved region in the EBL proteins is implicated in microneme targeting of the malaria parasite *Plasmodium falciparum*. J. Biol. Chem. **281**, 31995–32003

6 Treeck, M., Zacherl, S., Herrmann, S., Cabrera, A. L., Kono, M., Struck, N. S., Engelberg, K., Haase, S., Frischknecht, F., Miura, K. et al. (2009) Functional analysis of the leading malaria vaccine candidate AMA-1 reveals an essential role for the cytoplasmic domain in the invasion process. PLoS Pathog. **5**, e1000322

7 Grüring, C., Heiber, A., Kruse, F., Ungefehr, J., Gilberger, T.-W. and Spielmann, T. (2011) Development and host cell modifications of *Plasmodium falciparum* blood stages in four dimensions. Nat. Commun. **2**, 165

8 Lambros, C. and Vanderberg, J. P. (1979) Synchronization of *Plasmodium falciparum* erythrocytic stages in culture. J. Parasitol. **65**, 418–420

9 Blom, N., Gammeltoft, S. and Brunak, S. (1999) Sequence and structure-based prediction of eukaryotic protein phosphorylation sites. J. Mol. Biol. **294**, 1351–1362

Received 8 November 2012/18 March 2013; accepted 2 April 2013
Published as BJ Immediate Publication 2 April 2013, doi:10.1042/BJ20121694

© 2013 The Author(s)
The author(s) has paid for this article to be freely available under the terms of the Creative Commons Attribution Licence (CC-BY) (http://creativecommons.org/licenses/by/3.0/) which permits unrestricted use, distribution and reproduction in any medium, provided the original work is properly cited.
